# MicroRNA-195-5p Targets MYB to Regulate Proliferation and Malignant Metastasis in Triple-Negative Breast Cancer via PI3K/AKT/mTOR Signaling

**DOI:** 10.1155/tbj/7303173

**Published:** 2025-10-16

**Authors:** Kewei Tang, Site Bai, Qiang Zhou, Songlian Liu, Leilan Yin, Yajun Tong, Ling Long, Ludi Ou, Qinghua Yin

**Affiliations:** Department of Oncology, Yueyang Central Hospital, Yueyang, Hunan, China

**Keywords:** MicroRNA-195-5p, MYB, PI3K/AKT/mTOR signaling, proliferation, triple-negative breast cancer

## Abstract

**Objective:**

To investigate the effect of microRNA-195-5p (miRNA-195-5p) on proliferation and malignant metastasis in triple-negative breast cancer (TNBC) cells and its underlying mechanism.

**Methods:**

Expression levels of miRNA-195-5p and MYB were determined by quantitative real-time PCR (RT-qPCR) in TNBC cells (MDA-MB-231 and BT-549) and normal human mammary epithelial cells (MCF-10A). Cell proliferation was assessed via CCK-8 assays after miRNA-195-5p overexpression or knockdown in MDA-MB-231 cells. Transwell assays evaluated cellular invasion and migration. Western blotting analyzed impacts on the PI3K/AKT/mTOR pathway. Targeting of MYB by miRNA-195-5p was confirmed using TargetScan prediction and dual-luciferase reporter assays. RT-qPCR measured MYB expression upon miRNA-195-5p modulation. Rescue experiments (co-overexpression of MYB and miRNA-195-5p) further assessed proliferation and PI3K/AKT/mTOR signaling via CCK-8 and Western blotting.

**Results:**

Compared to MCF-10A cells, miRNA-195-5p expression was significantly downregulated (*p* < 0.01), while MYB was markedly upregulated (*p* < 0.001) in TNBC cells. Overexpression of miRNA-195-5p inhibited MDA-MB-231 proliferation, invasion, and migration; conversely, its knockdown promoted these phenotypes. MiRNA-195-5p directly targeted and negatively regulated MYB. MYB overexpression activated the PI3K/AKT/mTOR pathway, enhancing cell proliferation. Rescue experiments indicated that MYB upregulation counteracted the tumor-suppressive effects of miRNA-195-5p and reactivated PI3K/AKT/mTOR signaling.

**Conclusion:**

miRNA-195-5p suppresses proliferation and metastasis in TNBC by targeting MYB and inhibiting the PI3K/AKT/mTOR pathway.

## 1. Introduction

Triple-negative breast cancer (TNBC) represents a notably aggressive variant of breast cancer, comprising approximately 15%–20% of all breast cancer diagnoses [1–3]. This subtype is defined by the absence of estrogen receptors, progesterone receptors, and human epidermal growth factor receptor 2 (HER2), which results in a dismal prognosis and restricted treatment alternatives [4–6]. The absence of targeted therapeutic options for TNBC is associated with a heightened incidence of metastasis and recurrence, rendering it one of the most formidable forms of breast cancer to manage clinically [7–9]. Presently, the primary approach to clinical treatment involves chemotherapy, which, although initially effective, often engenders resistance and fails to tackle the fundamental molecular mechanisms that propel tumor progression [10, 11].

Recent investigations have underscored the significance of microRNAs (miRNAs) in the realm of cancer biology, particularly regarding their potential as therapeutic targets attributable to their regulatory role in gene expression [12–14]. Nevertheless, the specific dynamics through which individual miRNAs, such as miRNA-195-5p, affect TNBC progression remain insufficiently elucidated [15]. This knowledge deficit accentuates the necessity for further exploration into the regulatory networks encompassing miRNAs and their target genes, which could facilitate the development of innovative therapeutic strategies aimed at enhancing patient outcomes in TNBC.

miRNAs are innate noncoding RNAs (18–25 nucleotides) that modulate gene expression by means of mRNA cleavage or translational repression [16]. In TNBC, the dysregulation of miRNA is instrumental in promoting metastasis (miRNA-10b), chemoresistance (miRNA-221), and immune evasion (miRNA-146a) [17]. Notably, miRNA-195-5p (located on chr17p13.1) is part of the tumor-suppressive miRNA-15/16/195/424/497 family, characterized by the conserved seed sequence “AAGCAGC” [18]. MicroRNA-195-5p (miRNA-195-5p) is a diminutive noncoding RNA that is essential in regulating gene expression at the post-transcriptional stage [19]. It is recognized for its role in various biological processes, including cellular proliferation, apoptosis, and differentiation. In oncological contexts, miRNA-195-5p has been characterized as a putative tumor suppressor, with investigations revealing its capability to impede tumor growth and metastasis by targeting oncogenes [20–22]. In particular, miRNA-195-5p has been demonstrated to negatively modulate MYB, a transcription factor associated with the advancement of multiple malignancies [23].

Emerging evidence suggests that MYB is frequently overexpressed in TNBC, contributing to aggressive tumor characteristics and unfavorable patient prognoses [24]. The aberrant regulation of miRNA-195-5p and MYB in TNBC underscores a critical gap in the comprehension of the molecular mechanisms that drive tumorigenesis in this breast cancer subtype. Although the tumor-suppressive properties of miRNA-195-5p have been acknowledged, the precise pathways through which it exerts its effects, particularly concerning MYB and the PI3K/AKT/mTOR signaling cascade, remain inadequately investigated. Furthermore, the existing literature is deficient in comprehensive studies that explore these interactions.

The PI3K/AKT/mTOR signaling axis was selected for investigation due to its well-established role in promoting tumor growth, survival, and therapy resistance in TNBC. This pathway is frequently dysregulated in TNBC and contributes to its aggressive phenotype and poor clinical outcomes [25]. Previous studies have implicated MYB in the activation of oncogenic signaling pathways, including PI3K/AKT/mTOR, suggesting a potential mechanistic link between MYB overexpression and pathway activation [26, 27]. Additionally, miRNA-195-5p has been reported to target components of the PI3K/AKT/mTOR cascade in other cancer types, supporting its role as a key regulatory miRNA in this pathway [28]. Given these connections, we hypothesized that miRNA-195-5p may suppress TNBC progression by targeting MYB and subsequently inhibiting PI3K/AKT/mTOR signaling—an axis central to TNBC pathogenesis yet underexplored in the context of miRNA-195-5p and MYB regulation.

## 2. Methods

### 2.1. Cell Culture

The MDA-MB-231, BT-549, and MCF-10A cell lines were obtained from the Cell Bank of the Chinese Academy of Sciences (Shanghai, China). Cells were maintained in DMEM/F12 medium (Gibco, USA) supplemented with 10% fetal bovine serum (FBS; Gibco) and 1% penicillin–streptomycin at 37°C in a humidified atmosphere containing 5% CO_2_. Upon reaching 70%–80% confluence, the cells were passaged using 0.25% trypsin-EDTA, centrifuged at 800 × g for 5 min at room temperature, and resuspended in fresh complete medium. For transfection experiments, cells in the logarithmic growth phase were seeded into 24-well plates at a density of 2 × 10^5^ cells per well and incubated overnight.

### 2.2. Cell Transfection and Grouping

The miRNA-195-5p overexpression plasmid, miRNA-195-5p mimic (silencing construct), and corresponding negative controls (NCs) were synthesized by Wuhan Hengyisai Biotechnology Co., Ltd. Primer sequences were as follows:

  miRNA-195-5p mimic: Forward 5′-UAGCAGCACAGAAAUAUUGGC-3′, Reverse 3′-AUCGUCGUGUCUUUAUAACCG-5′
  miRNA-195-5p mimic NC: 5′-GCCAAUAUUUCUGUGCUGCUA-3′
  miRNA-195-5p inhibitor: Forward 5′-UUUGUACUACACAAAAGUACUG-3′, Reverse 3′-AAACAUGAUGUGUUUUCAUGAC-5′
  miRNA-195-5p inhibitor NC: 5′-CAGUACUUUUGUGUAGUACAAA-3′

Transfection was performed using Lipofectamine 2000 (Invitrogen, USA) in OptiMEM medium (Gibco) according to the manufacturer's protocol. Cells were transfected with 50 nM final concentrations of miRNA-195-5p mimic, miRNA-195-5p inhibitor, or their respective NC. Experimental groups included the following: control (untreated), miRNA-195-5p mimic NC, miRNA-195-5p mimic, miRNA-195-5p inhibitor NC, and miRNA-195-5p inhibitor. After 6 h, the medium was replaced with complete DMEM/F12 containing 10% FBS. Cells were harvested 48 h post-transfection for downstream assays.

### 2.3. CCK-8 Proliferation Assay

Transfected cells were seeded into 96-well plates (1 × 10^4^ cells/well) and cultured for 36 h. After 24 h of transfection, cell viability was assessed using the CCK-8 kit (Sigma-Aldrich, USA) according to the manufacturer's protocol. Absorbance at 450 nm was measured using a microplate reader, and proliferation rates were normalized to control groups.

### 2.4. Transwell Assay

Cell motility was evaluated using 24-well Transwell chambers (8 μm pore size, Corning) with serum gradient induction. For invasion assessment, polycarbonate membranes were precoated with 50 μL Matrigel matrix (1:8 dilution in serum-free medium, BD Biosciences) and polymerized at 37°C for 4 h. Migration assays utilized uncoated membranes.

Serum-starved cells (6 × 10^4^ cells/200 μL serum-free medium) were seeded in upper chambers, while complete medium containing 10% FBS served as chemoattractant in lower compartments. After 24-h incubation (37°C, 5% CO_2_), nonmigratory cells on the membrane's upper surface were removed by cotton swab abrasion. Transmembrane cells were fixed with 4% paraformaldehyde (15 min), stained with 0.1% crystal violet (20 min), and quantified through five randomized microscopic fields (200 ×, Olympus IX73).

### 2.5. Dual-Luciferase Reporter Assay for Detection of Luciferase Activity

Potential target genes of miRNA-195-5p were predicted using TargetScan. A wild-type MYB reporter plasmid (MYB wt) was constructed, and a mutant-type MYB reporter plasmid (MYB mut) was generated using a site-directed mutagenesis kit (ELK Biotechnology, China). Well-growing MDA-MB-231 cells were seeded into 12-well plates. When the cells reached 70%–75% confluence, MYB wt or MYB mut plasmids were co-transfected with either miRNA-195-5p mimics or miRNA-195-5p mimics NC into MDA-MB-231 cells using TurboFect transfection reagent. After 48 h, luciferase activity was measured using a Dual-Luciferase Reporter Assay Kit (Beyotime Biotechnology, Shanghai, China).

H-MYB primers:

  H-MYB Forward: 5′-CCGGTACCGCTAGCGGATCCGCATTACTCTAAGTGTAGAC-3′
  H-MYB Reverse: 5′-GCCGGCCGAGATCGAAGCTTGCGGATCAAATGCAGAACTC-3′

### 2.6. RT-qPCR

Total RNA was extracted using the ELK RNA kit (ELK Biotechnology, China) and reverse-transcribed into cDNA.

miRNA-195-5p primers:

  Forward: 5′-TGCGATGTAGCAGCACAGAA-3′
  Reverse: 5′-CTCAACTGGTGTCGTGGAGTCGGCAATTCAGTTGAGGCCAATAT-3′

MYB primers:

  Forward: 5′-CAAGTGAACTCTCACTCAACATACG-3′
  Reverse: 5′-CTTCATCATGGTGTGATCTCAGC-3′

U6 (internal control) primers:

  Forward: 5′-AACGCTTCACGAATTTGCGT-3′
  Reverse: 5′-CTCGCTTCGGCAGCACAT-3′

GAPDH (internal control) primers:

  Forward: 5′-CATCATCCCTGCCTCTACTGG-3′
  Reverse: 5′-GTGGGTGTCGCTGTTGAAGTC-3′

qPCR was performed using SYBR Green Master Mix (20 μL reaction: 10 μL 2 × SYBR Mix, 8 μL H2O, 0.5 μL each primer, 1 μL cDNA) under the following conditions: 95°C for 30 s, 40 cycles of 95°C/30 s and 60°C/30 s, followed by 72°C for 50 s. Relative expression was calculated using the 2 − ΔΔCt method.

### 2.7. Western Blot

Cells were lysed in RIPA buffer containing protease inhibitors. Protein concentrations were determined via BCA assay (Invitrogen). Samples (35 μg/lane) were separated by SDS-PAGE and transferred to PVDF membranes. After blocking with 5% nonfat milk, membranes were incubated overnight at 4°C with primary antibodies against p-PI3K (ab278545, Abcam), PI3K (ab302958, Abcam), p-AKT (ab38449, Abcam), AKT (ab8805, Abcam), p-mTOR (ab109268, Abcam), mTOR (ab134903, Abcam), or GAPDH (ab9485, Abcam). HRP-conjugated secondary antibodies were applied for 1 h at room temperature. Protein bands were visualized using ECL substrate and quantified with ImageJ.

### 2.8. Statistical Analysis

Data are presented as mean ± SD. Statistical comparisons were performed using GraphPad Prism 9.0 (one-way ANOVA for multiple groups, Student's t-test for pairwise comparisons). A threshold of *p* < 0.05 was considered statistically significant.

## 3. Results

### 3.1. Differential Expression of miRNA-195-5p in Breast Cell Lines and Its Successful Experimental Modulation

**Figure 1:**
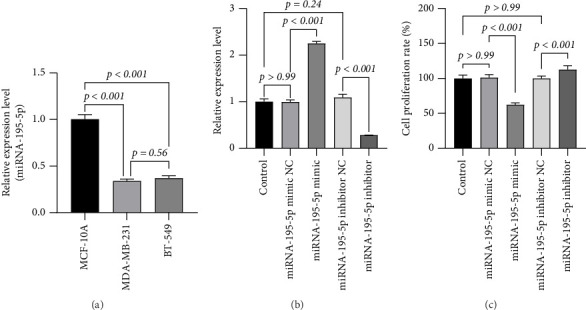
miRNA-195-5p exhibits tumor-suppressive properties in breast cancer cell lines. (a) Relative expression levels of miRNA-195-5p in the normal breast epithelial cell line (MCF-10A) and two breast cancer cell lines (MDA-MB-231 and BT-549). miRNA-195-5p expression was significantly downregulated in cancer cells compared to normal cells. (b) Validation of miRNA-195-5p overexpression efficiency. Cells were transfected with miRNA-195-5p mimic or negative control (mimic NC). The mimic group showed a significant increase in miRNA-195-5p expression compared to the control and mimic NC groups. (c) Cell proliferation assay. Ectopic expression of miRNA-195-5p (mimic) significantly inhibited the proliferation of breast cancer cells compared to controls (control and mimic NC groups). Data are presented as mean ± SD from three independent experiments. Representative of three experiments. The test was conducted using the one-way ANOVA test, and the *p*-values are presented in numerical form in the graph.

RT-qPCR analysis was performed to evaluate the endogenous expression pattern of miRNA-195-5p across different mammary cell lines, given its previously reported tumor-suppressive roles in certain cancers. Specifically, we compared its expression in the nontumorigenic mammary epithelial cell line MCF-10A with two TNBC cell lines, MDA-MB-231 and BT-549. This comparative approach was designed to determine whether miRNA-195-5p expression is associated with the malignant phenotype. The results revealed that miRNA-195-5p was highly expressed in MCF-10A cells, but its expression was significantly downregulated in both TNBC cell lines (*p* < 0.001). Notably, the most aggressive cell line, MDA-MB-231, exhibited the lowest expression level (Figure 1(a)), suggesting a potential inverse correlation between miRNA-195-5p expression and tumor aggressiveness.

To functionally characterize miRNA-195-5p, we next performed gain-of-function and loss-of-function experiments by transfecting MDA-MB-231 cells with either a miRNA-195-5p mimic or a specific inhibitor, using lipofectamine-based transfection under optimized conditions. Corresponding NCs for both mimic and inhibitor were included to account for non–sequence-specific transfection effects. The transfection efficiency was validated 48 h post-transfection using RT-qPCR. Compared with the untransfected control, cells transfected with the miRNA-195-5p mimic showed a 2.25-fold increase in expression (*p* < 0.001), whereas cells treated with the inhibitor exhibited a 72% reduction in transcript levels (*p* < 0.001). Importantly, neither the mimic NC nor the inhibitor NC groups showed significant changes in expression (*p* > 0.99 and *p*=0.24, respectively) (Figure 1(b)), confirming that the observed effects were specifically due to miRNA-195-5p modulation.

These findings confirmed the successful establishment of an in vitro model with altered miRNA-195-5p expression, which enabled us to proceed with subsequent functional assays to investigate its effects on malignant behaviors such as proliferation, migration, and invasion in TNBC models.

### 3.2. MiR-195-5p Suppresses Malignant Phenotypes of MDA-MB-231 Cells by Inhibiting Proliferation, Migration, and Invasion

To investigate the functional role of miRNA-195-5p in TNBC cell malignancy, we performed gain- and loss-of-function experiments in MDA-MB-231 cells using synthetic miRNA mimics and inhibitors, respectively. The experimental design included mimic/inhibitor transfection groups along with their corresponding NCs to rule out nonspecific oligonucleotide effects.

We first assessed the impact of miRNA-195-5p on cell proliferation using the CCK-8 assay. Ectopic expression of miRNA-195-5p significantly suppressed cellular viability, resulting in a 62.28% reduction compared to the mimic NC group (*p* < 0.001). Conversely, inhibition of endogenous miRNA-195-5p enhanced proliferative capacity by 13% (1.13-fold, *p* < 0.001). Both NC groups—mimic NC and inhibitor NC—showed no significant difference in proliferation relative to untreated controls (*p* > 0.99), confirming that the observed effects were specifically attributable to miRNA-195-5p modulation (Figure 1(c)). These findings indicate that miRNA-195-5p acts as a potent suppressor of MDA-MB-231 cell growth.

We next evaluated the role of miRNA-195-5p in metastatic behaviors using Transwell-based migration and invasion assays. To examine directional migration, cells were seeded into inserts with 8-μm pore membranes and allowed to migrate toward serum-containing medium. Overexpression of miRNA-195-5p markedly impeded cell motility, showing a 51.44% decrease relative to the mimic NC (*p* < 0.001). In contrast, miRNA-195-5p inhibition increased migration by 32% (1.32-fold, *p* < 0.001).

**Figure 2:**
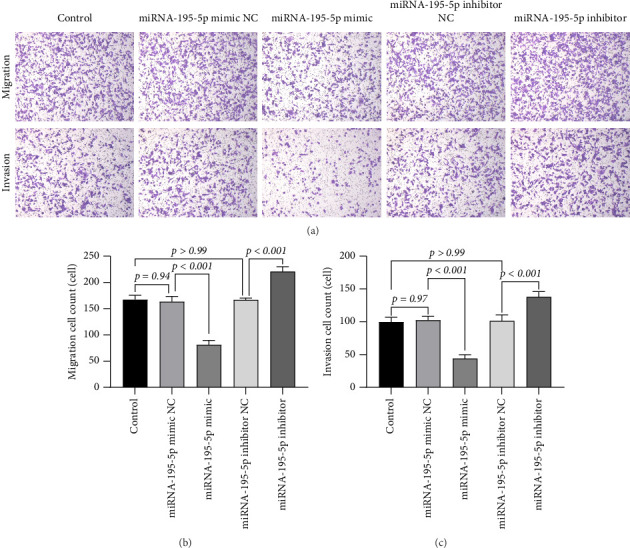
miRNA-195-5p suppresses migration and invasion of breast cancer cells MDA-MB-231. (a) Representative images of cell migration (upper panel) and invasion (lower panel) assays conducted using Transwell chambers. The five experimental groups from left to right are control, miRNA-195-5p mimic negative control (mimic NC), miRNA-195-5p mimic, miRNA-195-5p inhibitor negative control (inhibitor NC), and miRNA-195-5p inhibitor. Cells that migrated or invaded through the membrane are stained purple. (b and c) Quantitative analysis of migrated (b) and invaded (c) cells from the experiments shown in panel (a). Data are presented as the mean number of cells per field ± SD from three independent experiments. Statistical significance was determined by one-way ANOVA with Tukey's post hoc test.

To assess invasive potential, we employed Matrigel-coated Transwell inserts that simulate basement membrane penetration. Similarly, miRNA-195-5p mimic transfection resulted in a 57.03% reduction in invasiveness (*p* < 0.001), while inhibitor treatment enhanced invasion by 36% (1.36-fold, *p* < 0.001). Again, both NC groups exhibited baseline migration and invasion capacities (mimic NC: *p*=0.94 for migration, *p*=0.97 for invasion; inhibitor NC: *p* > 0.99 for both), confirming the specificity of the oligonucleotide effects (Figures 2(a), 2(b), 2(c)).

Notably, the suppressive effect of miRNA-195-5p was more pronounced in invasion than in migration, suggesting a particularly critical role in regulating matrix degradation and penetrative capability. Taken together, these results demonstrate that miRNA-195-5p functions as a bidirectional regulator of metastatic progression in MDA-MB-231 cells, exerting robust inhibition across multiple malignant phenotypes.

### 3.3. miRNA-195-5p Suppresses the Activation of PI3K/AKT/mTOR Signaling Pathway in MDA-MB-231 Cells

Based on our functional findings that miRNA-195-5p significantly inhibited proliferation, migration, and invasion, we sought to investigate the potential molecular mechanisms underlying these effects. Given the well-established role of the PI3K/AKT/mTOR signaling axis in promoting oncogenic phenotypes in breast cancer, we hypothesized that miRNA-195-5p might exert its tumor-suppressive functions by modulating this pathway. To test this, we employed Western blot analysis to evaluate both the phosphorylation status (activation) and total protein expression of key components within this pathway following miRNA-195-5p modulation.

MDA-MB-231 cells were transfected with miRNA-195-5p mimic, inhibitor, or their respective NCs. Whole-cell lysates were subsequently harvested and probed with antibodies specific for total and phosphorylated forms of PI3K, AKT, and mTOR. Densitometric analysis was performed to quantify protein levels, normalized to β-actin loading control.

**Figure 3:**
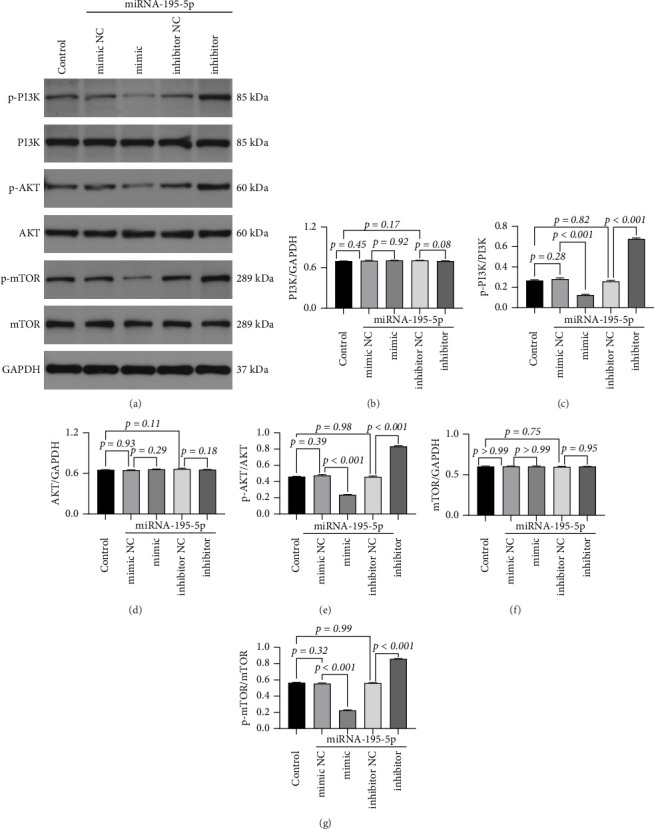
miRNA-195-5p inhibits the PI3K/AKT/mTOR signaling pathway in breast cancer cells MDA-MB-231. (a) Representative Western blot images showing the protein expression levels of phosphorylated and total PI3K, AKT, and mTOR in different treatment groups. Cells were transfected with: control (untreated), negative control for miRNA-195-5p mimic (mimic NC), miRNA-195-5p mimic, negative control for miRNA-195-5p inhibitor (inhibitor NC), and miRNA-195-5p inhibitor. GAPDH was used as a loading control. The molecular weight (in kDa) of each protein is indicated. (b–g) Densitometric quantification of the protein levels from panel (a). The expression levels of p-PI3K/PI3K (b), p-AKT/AKT (c), and p-mTOR/mTOR (d) were normalized to their respective total proteins. The total protein levels of PI3K (e), AKT (f), and mTOR (g) were normalized to GAPDH. Data are presented as mean ± SD (*n* = 3 independent experiments). Statistical significance was determined by one-way ANOVA with Tukey's post hoc test. Data are represented as mean ± SD. Representative of three experiments. The test was conducted using the one-way ANOVA test, and the *p*-values are presented in numerical form in the graph.

Our results demonstrated that miRNA-195-5p specifically regulates the phosphorylation and activation of these kinases without altering their total protein abundance. Transfection with the miRNA-195-5p mimic led to a profound suppression of pathway activation, evidenced by significant reductions in the levels of phosphorylated PI3K (p-PI3K, down 55.89%, *p* < 0.001), phosphorylated AKT (p-AKT, down 50.60%, *p* < 0.001), and phosphorylated mTOR (p-mTOR, down 59.47%, *p* < 0.001) compared to its NC group. Conversely, inhibiting endogenous miRNA-195-5p with a specific antagonist enhanced pathway activation, resulting in increased levels of p-PI3K (2.58-fold, *p* < 0.001), p-AKT (1.79-fold, *p* < 0.001), and p-mTOR (1.53-fold, *p* < 0.001) relative to its NC. Critically, no significant changes were observed in the total protein expression of PI3K, AKT, or mTOR across any treatment groups (*p* > 0.05). Furthermore, the phosphorylation levels in both NC groups remained statistically unchanged from the baseline (mimic NC: *p* = 0.573–0.954; inhibitor NC: *p* = 0.125–0.944), effectively excluding any nonspecific effects of the transfection reagents or oligonucleotides themselves (Figure 3).

These data infer that miRNA-195-5p acts as a potent upstream regulator that dampens the oncogenic PI3K/AKT/mTOR signaling cascade by inhibiting the phosphorylation of its core components. This targeted suppression of pathway activation provides a plausible mechanistic explanation for the observed reductions in cell proliferation, migration, and invasion. The finding that miRNA-195-5p does not affect total protein levels suggests it may directly target upstream positive regulators of this pathway or the kinases themselves. This insight directs the next step of our investigation: to identify and validate the direct mRNA targets of miRNA-195-5p that mediate this specific inhibitory effect on PI3K/AKT/mTOR signaling.

### 3.4. miRNA-195-5p Directly Targets and Suppresses MYB Oncogene Expression in TNBC Cells

**Figure 4:**
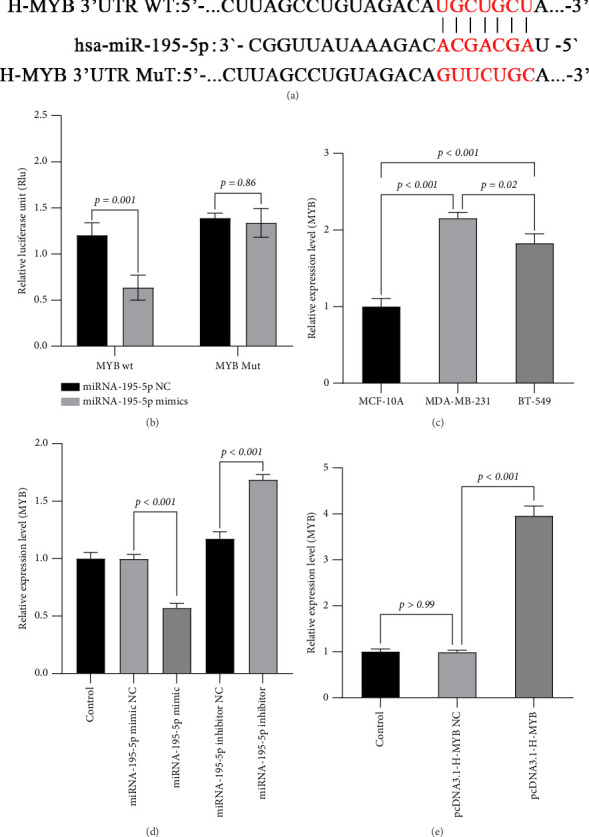
miR-195-5p directly targets and suppresses MYB expression. (a) Schematic representation of the predicted binding site for hsa-miR-195-5p within the 3′-untranslated region (3′UTR) of MYB mRNA. The seed sequence complementarity is highlighted in red. Mutations (MuT) were introduced into the MYB 3′UTR to disrupt miR-195-5p binding. (b) Luciferase reporter assay. MDA-MB-231 cells were co-transfected with either the wild-type (WT) or mutant (MuT) MYB 3′UTR reporter plasmid, along with miR-195-5p mimics or negative control (NC). Firefly luciferase activity was normalized to Renilla luciferase activity. Data are presented as relative luciferase activity (mean ± SD, *n* = 3). Statistical significance was determined by a two-way ANOVA with Sidak's post hoc test. (c–e) Relative mRNA expression levels of MYB in (c) MDA-MB-231, (d) BT-549, and (e) MCF-7 cells following transfection with miR-195-5p mimics or inhibitor and their respective negative controls (NCs). Gene expression was normalized to GAPDH. Data are presented as mean ± SD from three independent experiments. Statistical analysis was performed using an unpaired t-test.

To elucidate the molecular mechanism by which miRNA-195-5p exerts its tumor-suppressive effects, we sought to identify its direct downstream targets. Based on our previous findings that miRNA-195-5p inhibits the PI3K/AKT/mTOR pathway and malignant phenotypes, we hypothesized that it might target key oncogenic regulators. Through bioinformatic analysis using the TargetScan algorithm, we identified MYB, a well-characterized oncogene in breast cancer, as a top candidate due to the presence of a highly conserved binding site for miRNA-195-5p in its 3′ untranslated region (3′UTR) (Figure 4(a)).

To experimentally validate this prediction, we performed dual-luciferase reporter assays. We cloned the wild-type (wt) MYB 3′UTR sequence containing the predicted miRNA-195-5p binding site downstream of a firefly luciferase gene. A mutant (mut) construct with substitutions in the seed-binding region was also generated as a control. Co-transfection of MDA-MB-231 cells with miRNA-195-5p mimic and the wt reporter plasmid resulted in a significant reduction (*p*=0.001) in luciferase activity compared to the mimic NC group. In contrast, the mutant reporter construct was completely resistant to miRNA-195-5p-mediated suppression (*p*=0.86), demonstrating the specificity of this interaction and confirming MYB as a direct target of miRNA-195-5p (Figure 4(b)).

We next examined whether miRNA-195-5p-mediated regulation of the MYB 3′UTR translates to changes in endogenous MYB expression. Quantitative RT-PCR analysis revealed that ectopic expression of miRNA-195-5p mimic significantly downregulated MYB mRNA levels compared to both untreated controls and mimic NC-transfected cells (*p* < 0.001). Conversely, inhibition of endogenous miRNA-195-5p with a specific antagonist increased MYB expression compared to inhibitor NC groups (all *p* < 0.01) (Figure 4(d)). These results establish a reciprocal regulatory relationship where miRNA-195-5p negatively regulates MYB expression.

Given this targeting relationship, we investigated the clinical relevance of MYB expression in breast cancer progression. We compared MYB expression levels across cell lines and found significantly elevated MYB expression in TNBC cells (MDA-MB-231 and BT-549) compared to normal breast epithelial cells (MCF-10A) (*p* < 0.001). Among the TNBC lines, MDA-MB-231 cells exhibited the highest MYB expression (*p*=0.02 versus BT-549), suggesting a potential correlation between MYB overexpression and aggressive cancer phenotypes (Figure 4(c)).

Collectively, these findings demonstrate that MYB is a direct functional target of miRNA-195-5p in MDA-MB-231 cells. The inverse relationship between miRNA-195-5p (frequently downregulated in cancers) and its target MYB (frequently upregulated) provides a mechanistic explanation for our observed phenotypic effects—whereby loss of miRNA-195-5p in TNBC leads to MYB overexpression, consequently driving proliferation, migration, and invasion through activation of oncogenic signaling pathways. These results position the miRNA-195-5p/MYB axis as a critical regulatory node in TNBC pathogenesis and suggest that therapeutic restoration of miRNA-195-5p could suppress MYB-driven oncogenesis.

### 3.5. MYB Overexpression Rescues miRNA-195-5p-Mediated Suppression of Proliferation and PI3K/AKT/mTOR Signaling

To establish whether MYB is a functional downstream effector through which miRNA-195-5p exerts its tumor-suppressive effects, we performed a rescue experiment by overexpressing MYB in the context of miRNA-195-5p upregulation. MDA-MB-231 cells were first transfected with miRNA-195-5p mimic to suppress endogenous MYB, followed by transfection with a lentiviral MYB overexpression vector (pcDNA3.1(+) linearized vector) or empty vector control (OE-NC). Quantitative analysis confirmed successful MYB overexpression, with significantly elevated MYB levels in the OE-MYB group compared to both the control and OE-NC groups (*p* < 0.001; Figure 4(e)).

**Figure 5:**
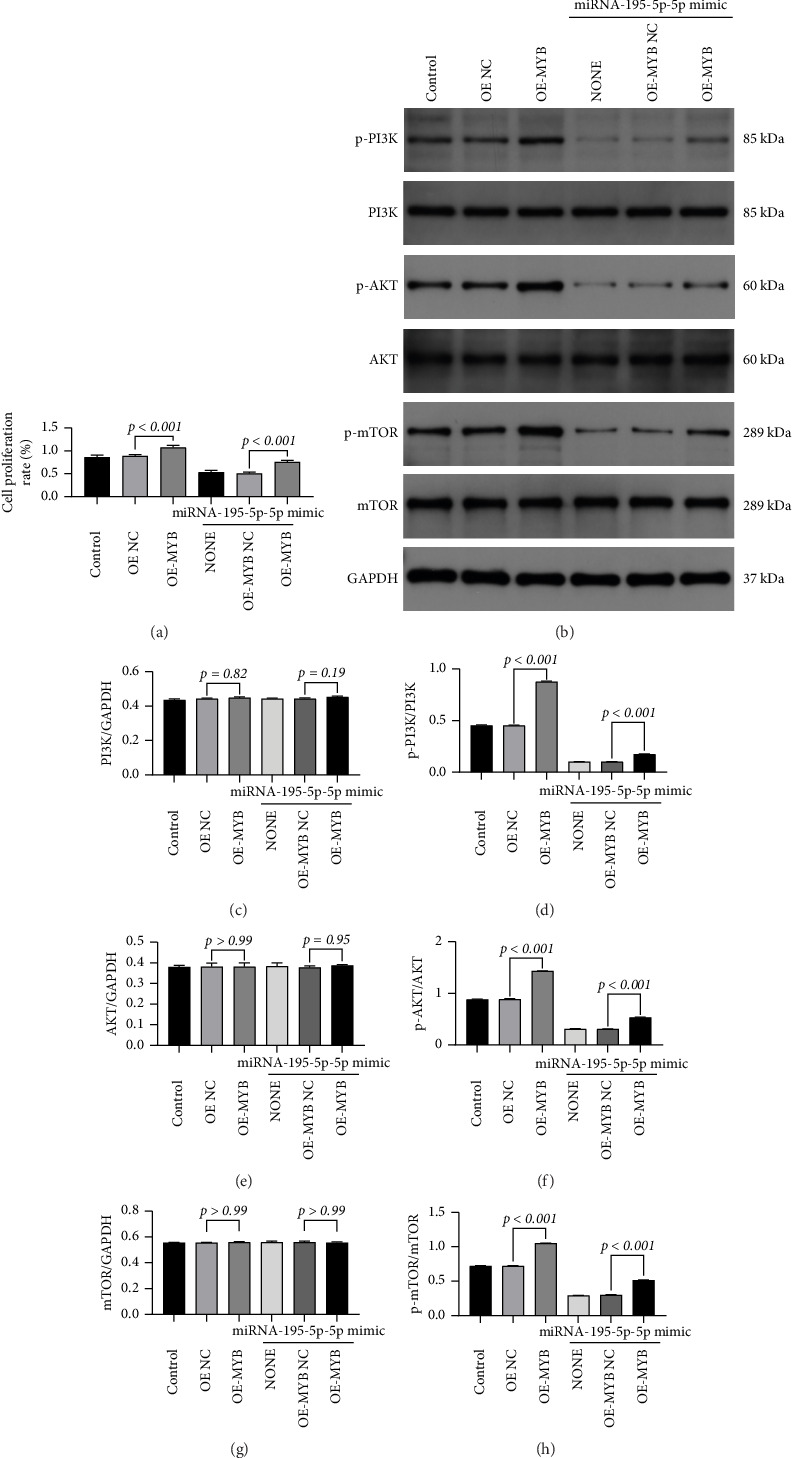
Overexpression of MYB rescues the tumor-suppressive effects of miR-195-5p by restoring PI3K/AKT/mTOR signaling activity. (a) Cell proliferation assay. Overexpression of MYB (OE-MYB) significantly rescued the proliferation inhibition induced by miRNA-195-5p mimic in breast cancer cells MDA-MB-231. The groups are as follows: control, negative control for MYB overexpression (OE-NC), MYB overexpression (OE-MYB), miRNA-195-5p mimic, and miRNA-195-5p mimic combined with MYB overexpression (miRNA-195-5p mimic + OE-MYB). (b) Representative Western blot images showing the expression levels of MYB and key proteins in the PI3K/AKT/mTOR signaling pathway, along with their phosphorylation states, under the same treatment conditions as in (a). GAPDH serves as a loading control. (c–h) Densitometric quantification of the protein levels from panel (b). The expression levels of MYB (c) and the ratios of p-PI3K/PI3K (d), p-AKT/AKT (e), and p-mTOR/mTOR (f) were assessed. The total protein levels of PI3K (g) and AKT (h) were also analyzed. Data were normalized to GAPDH (for total protein) or to their respective total proteins (for phosphorylation ratios)

Using this rescue system, we first assessed cell proliferation via the CCK-8 assay. As expected, miRNA-195-5p mimic alone significantly suppressed proliferation compared to the OE-NC group (*p* < 0.001; Figure 5(a)). Conversely, MYB overexpression alone enhanced proliferative capacity (*p* < 0.001). Importantly, subsequent MYB overexpression largely reversed the antiproliferative effect caused by miRNA-195-5p mimic (*p* < 0.001 versus mimic alone group; Figure 5(a)).

We next examined whether MYB-mediated rescue occurred through reactivation of the PI3K/AKT/mTOR pathway. Western blot analysis demonstrated that miRNA-195-5p mimic significantly reduced phosphorylation levels of PI3K, AKT, and mTOR, as well as the p-PI3K/PI3K, p-AKT/AKT, and p-mTOR/mTOR ratios (all *p* < 0.001 versus OE-NC; Figures 5(b), 5(c), 5(d), 5(e), 5(f), 5(g), 5(h)). MYB overexpression alone produced the opposite effect, markedly increasing both phosphorylation levels and phosphorylation ratios of all three pathway components (all *p* < 0.001). Critically, MYB overexpression in miRNA-195-5p-overexpressing cells effectively restored phosphorylation levels and ratios to near baseline, significantly reversing the suppression induced by miRNA-195-5p mimic (all *p* < 0.001 versus mimic alone group; Figures 5(b), 5(c), 5(d), 5(e), 5(f), 5(g), 5(h)).

These rescue results provide compelling functional evidence that MYB operates downstream of miRNA-195-5p to regulate both cellular proliferation and PI3K/AKT/mTOR pathway activation. The ability of ectopic MYB expression to counteract the phenotypic and molecular effects of miRNA-195-5p overexpression establishes a direct causal relationship within this regulatory axis. These findings solidify the mechanistic pathway whereby miRNA-195-5p exerts its tumor-suppressive effects in TNBC by targeting MYB, consequently inhibiting PI3K/AKT/mTOR signaling and cellular proliferation.

## 4. Discussion

Understanding the intricate regulatory mechanisms of miRNAs in cancer progression is essential [29, 30]. This knowledge is pivotal for identifying and developing novel therapeutic strategies to combat this pervasive disease. This study has several advantages, including the innovative use of multiple complementary methods to assess the functional impact of miRNA-195-5p on TNBC cells. The key results from this research demonstrate that miRNA-195-5p is notably downregulated in TNBC and that it negatively regulates the expression of MYB, a critical oncogene. Furthermore, the findings reveal that MYB overexpression counteracts the tumor-suppressive effects of miRNA-195-5p and activates the PI3K/AKT/mTOR signaling pathway, which promotes cancer progression. This leads to activation of the PI3K/AKT/mTOR signaling pathway, which plays a crucial role in cell growth and survival and ultimately contributes to cancer progression.

Our observation of reduced intracellular miRNA-195-5p levels in TNBC cells is consistent with multiple studies reporting its downregulation in breast cancer tissues. Specifically, miRNA-195 is suppressed in breast tumors regardless of histological grade, nodal status, or TNM staging, with this suppression being more pronounced in triple-negative and luminal subtypes [31]. This pattern aligns with its characterized role as a tumor suppressor miRNA across multiple cancer types [32–35]. Interestingly, while we observed intracellular downregulation of miRNA-195-5p in TNBC cells, previous studies have reported elevated circulating miRNA-195 levels in plasma samples from TNBC patients [36]. This apparent discrepancy may be explained by the proposed mechanism wherein cancer cells selectively export tumor suppressor miRNAs such as miRNA-195 to maintain oncogenesis. This active export mechanism would simultaneously reduce intracellular levels (thereby relieving target gene repression) while increasing extracellular levels in circulation [36]. The consistent downregulation of miRNA-195-5p in breast cancer tissues suggests it may serve as a potential diagnostic biomarker. Studies have shown that miRNA-195 expression levels in tumor tissues can differentiate malignant from normal breast tissue, and its expression may be modulated by therapeutic interventions [37]. Furthermore, in Turkish breast cancer patients, decreased miRNA-195 levels were observed in postoperative blood samples compared to preoperative levels, suggesting dynamic changes associated with disease status [31]. The functional significance of miRNA-195-5p downregulation in TNBC likely extends beyond any single target gene, as this miRNA has been shown to regulate multiple oncogenic pathways. In other cancer contexts, miRNA-195-5p has been demonstrated to inhibit cell proliferation, migration, and invasion through regulation of various target genes and pathways [32, 34, 35]. The specific consequences of its downregulation in TNBC warrant further investigation to fully elucidate its tumor-suppressive mechanisms in this aggressive breast cancer subtype.

The significant upregulation of MYB in TNBC cells suggests its potential as a therapeutic target, consistent with observations in adenoid cystic carcinoma (ACC), where MYB inhibition via peptidomimetics suppresses tumor growth [38]. MYB's role in cancer biology is context-dependent: It drives proliferation in malignancies like colorectal cancer and embryonal rhabdomyosarcoma [39, 40], yet paradoxically enhances antitumor immunity in immunogenic tumors (e.g., colon carcinoma) by activating cytotoxic T cells [41]. Structural alterations (e.g., truncations or alternative splicing) may further potentiate its oncogenicity [42, 43]. Thus, while MYB represents a tractable target for TNBC therapy, its dual functionalities—especially potential immunomodulatory effects—warrant careful evaluation to mitigate unintended consequences during intervention.

Emerging experimental evidence demonstrates that miRNA-195 functions as a tumor suppressor by directly targeting MYB, as validated in cervical cancer models where its overexpression represses MYB at transcriptional and translational levels, consequently inhibiting proliferation, migration, and invasion [35]. This regulatory axis may extend to breast cancer, supported by elevated circulating miRNA-195-5p in breast cancer patients. The context-dependent duality of miRNA-195-5p acting as either oncogene or tumor suppressor across malignancies underscores its therapeutic potential for aggressive subtypes like TNBC [44]. While the current literature primarily evidences this regulation in cervical cancer, our preliminary experimental data indeed reveal a targeting and negative regulatory relationship between miRNA-195-5p and MYB in TNBC cell lines. These findings collectively highlight miRNA-mediated oncogene regulation as a promising avenue for targeted therapies.

The PI3K/AKT/mTOR signaling axis was chosen for investigation in this study due to its well-recognized role in regulating cell proliferation, survival, and therapy resistance in cancer, particularly in aggressive breast cancer subtypes, such as TNBC and HER2+ breast cancer [25]. This pathway is frequently dysregulated in breast cancer and has been implicated in various resistance mechanisms, including those associated with trastuzumab treatment in HER2+ disease. Clinical observations have consistently shown that trastuzumab resistance often correlates with upregulation of key components within this pathway, such as AKT3 and MAP2K1, highlighting its relevance to compensatory signaling networks [45]. In our study, the selection of this axis was further motivated by the initial findings that MYB overexpression activates PI3K/AKT/mTOR signaling to enhance TNBC cell proliferation, which aligns with these broader clinical patterns. Additionally, the modulation of this pathway by miRNA-195-5p—through direct targeting of MYB—was supported by evidence of reduced plasma miRNA-195-5p levels in trastuzumab-resistant HER2+ patients exhibiting MYB overexpression. Rescue experiments confirmed the functional reciprocity, wherein MYB overexpression reversed the tumor-suppressive effects of miRNA-195-5p and reactivated PI3K/AKT/mTOR signaling, mirroring the clinical scenario of pathway activation during resistance. These collective insights underscore the rationale for focusing on this signaling axis and reinforce the potential of co-targeting MYB and miRNA-195-5p to overcome resistance mechanisms.

The current investigation has several limitations that warrant acknowledgment. Firstly, the reliance solely on in vitro models means that the functional significance of the miRNA-195-5p/MYB/PI3K/AKT/mTOR axis remains unvalidated in a physiologically relevant in vivo context. Future studies should prioritize the use of animal models, such as xenograft or genetically engineered mouse models, to confirm these findings and assess the axis's role in tumor progression and metastasis. Secondly, the sample size used in this study is relatively small, which may limit the generalizability and robustness of the results. To address this, larger, independent patient cohorts should be analyzed, and a meta-analysis of existing genomic datasets could be conducted to reinforce the conclusions across diverse populations. Thirdly, the absence of correlation analyses with clinical parameters—such as disease stage, treatment response, or overall survival—hinders the ability to evaluate the prognostic or predictive value of miRNA-195-5p expression in TNBC patients. Incorporating such clinical data in future work would enhance the translational relevance. Finally, potential technical variations, including batch effects from data collection across different platforms or time points, could introduce confounding factors; implementing standardized protocols and statistical adjustments (e.g., normalization methods) in subsequent studies would help mitigate these issues.

MYB (MYB proto-oncogene) is a transcription factor known to play a role in various cancers, including TNBC, where it often contributes to cell proliferation, survival, and therapy resistance. In the context of this study, MYB is positioned as a key mediator in the miRNA-195-5p-regulated pathway, potentially linking it to the PI3K/AKT/mTOR signaling axis, which is critical for tumor growth and metabolism. The relevance of MYB stems from its potential as a therapeutic target; for instance, its downregulation by miRNA-195-5p may suppress oncogenic signals, offering a novel avenue for intervention. Future experimentation should focus on validating MYB's role through functional assays, such as using siRNA or CRISPR-based knockdown of MYB in TNBC cell lines to observe effects on proliferation, apoptosis, and pathway activation. Additionally, in vivo experiments targeting MYB—e.g., with small molecule inhibitors or gene therapy approaches—could explore its utility in combination therapies, potentially improving outcomes for TNBC patients. This would not only clarify the mechanistic insights but also bridge the gap between basic research and clinical applications.

## 5. Conclusion

In conclusion, this study identifies miRNA-195-5p as a key tumor suppressor in TNBC, functioning through the downregulation of MYB and consequential inhibition of the PI3K/AKT/mTOR signaling pathway. The results establish a critical mechanistic link and provide a fundamental molecular basis for the dysregulation observed in TNBC. Despite the limitations inherent in this initial investigation, these findings illuminate the significant potential of leveraging miRNA-195-5p, either directly as a therapeutic agent or as a guide for drug discovery, in the development of novel, more effective treatment strategies tailored for TNBC. Further research, particularly focused on in vivo validation, clinical correlation, and exploration of delivery mechanisms for miRNA-based therapeutics, is warranted to translate these promising mechanistic insights into tangible clinical benefits for TNBC patients.

## Data Availability

The datasets generated during or analyzed during the current study are available from the corresponding author upon reasonable request.
